# Cardiovascular risks in subjects exposed to the Brumadinho dam collapse, Minas Gerais, Brazil

**DOI:** 10.6061/clinics/2019/e1301

**Published:** 2019-10-23

**Authors:** Vitor Engrácia Valenti, David M. Garner

**Affiliations:** I Centro de Estudos do Sistema Nervoso Autonomo, Faculdade de Filosofia e Ciencias, Universidade Estadual Paulista, Marilia, SP, BR; II Cardiorespiratory Research Group, Department of Biological and Medical Sciences, School of Health and Life Sciences, Oxford Brookes University, Headington Campus, Gipsy Lane, Oxford OX3 0BP, United Kingdom

On January 25^th^, 2019, structural problems with the Dam in Brumadinho, MG, Brazil caused serious fatalities for numerous families. It was responsible for 99 registered deaths and over 250 individuals disappeared, including employees and local residents (1,2). According to a survey by the Oswaldo Cruz Foundation of Brazil, the area concerned affects many kilometers of the Paraopeba River (3). The likelihood of an outbreak of diseases has already been highlighted, including yellow and dengue fevers, leptospirosis and schistosomiasis. Moreover, the short-term effects of heavy metal exposure (for example, aluminum, manganese and iron) are related to symptoms such as dizziness, diarrhea, nausea and vomiting because of the impact on their central nervous systems (4).

As follows, heavy metal exposure can happen in two ways: (a) oral intake; or (b) respiratory exposure via the nasal cavities. By oral intake, heavy metals reach the bloodstream after passing through the gastrointestinal tract (5). When the exposure is through the respiratory pathways, heavy metals enter the bloodstream via the contact of the tiny alveoli with minute blood vessels; to facilitate heavy metal deposits in the bloodstream (6). Within the blood circulation, heavy metals cause increased oxidative stress and lipid peroxidation at the cellular level (7).

Thus, a Japanese study (8) has revealed that heavy metal exposure is responsible for malignant pathophysiological responses to the cell. Amongst these effects, impairments of the blood vessels smooth muscle were detected, enabling the progression of vascular diseases.

Another study conducted in the United States of America (9) has revealed that blood vessels are a serious target for heavy metal exposure toxicity. Furthermore, it has been revealed that the consequences of heavy metals on blood vessels can play key roles in mediating the physio-pathological effects on different body parts; for example the kidneys, lungs and liver. Such exposure compromises the working of these structures.

In 2014, data collected by the Houston Methodist Research Institute (10) demonstrated there is compelling evidence connecting heavy metal toxicity to neuronal dysfunction. In this way, the neurons involvement influences the cardiovascular reflexes, contributing to the development of diseases such as hypertension, cardiac arrhythmias and strokes.

Recently, researchers at NC State University, Raleigh, North Carolina, USA (11) have cited 36 epidemiological studies and shown the negative impact of exposure to heavy metals on the development of Metabolic syndrome; which incorporates diabetes, dyslipidemia, obesity, and hypertension.

So, the references cited indicate a greater degree of risk for the progression of cardiovascular diseases in those exposed to heavy metals in the Brumadinho, MG, Brazil region and, in the vicinity of the Paraopeba River. We seize this opportunity to draw attention to the unfortunate persons in these situations. Critically, their cardiovascular fitness needs monitoring regularly.
